# Femtosecond Laser Cutting of 110–550 µm Thickness Borosilicate Glass in Ambient Air and Water

**DOI:** 10.3390/mi14010176

**Published:** 2023-01-10

**Authors:** Edgaras Markauskas, Laimis Zubauskas, Gediminas Račiukaitis, Paulius Gečys

**Affiliations:** Center for Physical Sciences and Technology, Savanoriu Ave. 231, LT-02300 Vilnius, Lithuania

**Keywords:** femtosecond pulses, cutting, roughness, chipping, characteristic strength, borosilicate glass, filament

## Abstract

The cutting quality and strength of strips cut with femtosecond-duration pulses were investigated for different thicknesses of borosilicate glass plates. The laser pulse duration was 350 fs, and cutting was performed in two environments: ambient air and water. When cutting in water, a thin flowing layer of water was formed at the front surface of the glass plate by spraying water mist next to a laser ablation zone. The energy of pulses greatly exceeded the critical self-focusing threshold in water, creating conditions favorable for laser beam filament formation. Laser cutting parameters were individually optimized for different glass thicknesses (110–550 µm). The results revealed that laser cutting of borosilicate glass in water is favorable for thicker glass (300–550 µm) thanks to higher cutting quality, higher effective cutting speed, and characteristic strength. On the other hand, cutting ultrathin glass plates (110 µm thickness) demonstrated almost identical performance and cutting quality results in both environments. In this paper, we studied cut-edge defect widths, cut-sidewall roughness, cutting throughput, characteristic strength, and band-like damage formed at the back surface of laser-cut glass strips.

## 1. Introduction

The use of femtosecond (fs) pulses has drastically increased over recent years in the processing of brittle transparent materials, such as scribing and cutting [1], femtosecond laser-induced selective etching [2], optical waveguide writing [3,4], and high-density optical storage formation [4,5]. 

The short duration of the femtosecond laser pulse is advantageous for its reduced thermal accumulation effects and nonlinear absorption compared to longer-duration pulses [1,6,7]. The electron–phonon coupling in dielectrics is usually longer than the fs pulse duration, allowing the delivery of the pulse energy quicker than the thermal diffusion occurs—energy transfer to surrounding material via phonon vibrations [8,9]. As a result, heat-accumulation-related stresses can be reduced and, together with higher energy absorption, can be confined to a smaller volume, improving the quality and strength of machined glass parts [9,10,11]. 

The use of femtosecond laser pulses allows the application of a wide variety of glass-cutting approaches, each providing its advantages and drawbacks. The most common laser-based cutting techniques employing femtosecond pulses are top-down cutting via direct laser ablation [12], bottom-up ablation [7,13], and laser stealth dicing [14,15].

Significant cutting speeds can be achieved via the stealth dicing technique. Mishchik et al. [14] reported a straight-line cutting speed of 600 mm/s of 500 µm thickness Eagle XG glass at a 100 kHz pulse repetition rate with fs duration pulses. A laser beam was focused inside the workpiece and scanned in a predetermined trajectory to form a stress layer. Usually, multifocal or Bessel beam processing is employed to improve cutting quality by elongating the modifications [16]. After the laser process, the workpiece is cleaved along the scanned line by applying mechanical stress. However, this technique is unsuitable for cutting tiny pieces or complex trajectories with a small radius of curvature (1 mm or less). Thus, it is mainly used for cutting straight lines [15]. 

The bottom-up approach does not have as strict limitations on cutting geometry as the stealth dicing technique while maintaining moderate glass-cutting speed [17,18]. A cutting speed of 9 mm/s of 1 mm thickness soda–lime glass sheet with nanosecond laser pulses was reported in [19]. Kerf formation starts from the back surface of the glass workpiece and gradually approaches the front surface. High cutting rates are reached due to the mechanical material removal nature since laser energy is used to crack the glass into small pieces instead of evaporating the material (a more energy-efficient approach than material ablation). However, the technique suffers from excessive cracking and chipping at the cut edges, with defects reaching up to hundreds of micrometers in size [13,18].

Finally, the direct laser ablation technique is based on the top-down material removal approach, where material ablation starts at the top of the workpiece and ends at the bottom by removing material in a layer-by-layer fashion. This approach allows the cutting of complex shapes consisting of inner and outer contours, coated, opaque, or highly absorptive materials [20]. This approach provides high process flexibility but suffers from low processing speeds and is unsuitable for taper-less cutting [18,21].

In the case of direct laser ablation, slow material cutting speeds can be compensated for by increasing the incident laser power. However, this leads to undesirable heat accumulation in the workpiece, which cannot be avoided even when femtosecond pulses are employed—especially when high pulse repetition rates are increasingly adopted in laser microfabrication [22,23]. Thus, despite the advantages provided by ultrashort femtosecond pulses, multiple undesirable effects could take place in brittle materials during laser–matter interaction: chipping [12,24], surface and subsurface micro-cracking [8,25], refractive index changes [26,27], electronic damage [28], and void formation [25,29]. Ultimately, laser-induced damage (defects) could cause the degradation of the cut-edge strength [7] and resistance to wear and tear, and negatively affect the longevity of laser-cut glass products.

Laser machining of glasses (and other brittle materials) can be conducted in liquids, most commonly in water, to further diminish the detrimental heat accumulation in the materials. In some cases, volatile liquids (ethanol, methanol, ethylene glycol, and others) are used in laser machining to cool down the workpiece [30,31,32]. However, Kanitz et al. [33] reported the highest specific ablation rate (μm^3^/μJ per laser shot) of iron in water, compared to ablation in methanol, ethanol, acetone, or toluene, while Liu et al. [34] observed deeper craters ablated in silicon when the workpiece was submerged in water than in ethanol. 

Previously, we demonstrated an improved cut-edge quality for borosilicate glass cutting in water with picosecond pulses [20,35]. Characteristic strength measurements in [35] revealed increased front and back side strengths by 7.2 and 10.9%, respectively, compared to cutting in ambient air. 

A comprehensive comparison of multiple laser-based glass-cutting techniques was conducted by Dudutis et al. [19,36]. They compared glass cutting via the bottom-up technique with direct laser ablation in ambient air and water [36] and stealth dicing [19]. In both studies, 1 mm thick soda–lime glass plates were used as the samples. Their findings revealed that in the case of 1064 nm radiation and picosecond (ps) duration pulses, the glass samples cut via direct laser ablation had the smallest sidewall roughness and possessed the highest front side cut-edge quality (smallest defect widths compared to the other two techniques). Furthermore, glass samples cut in water via direct ablation had the highest flexural strength (134 MPa at the front and 131 MPa at the back) compared to other investigated laser-cutting techniques. However, cutting of 1 mm thick soda–lime glass via direct laser ablation was notably slower (0.19 mm/s in ambient air and 0.34 mm/s in water) than the stealth dicing with Bessel beams (100 mm/s) and bottom-up cutting (0.74 mm/s) using 1064 nm wavelength ps pulses [19,36]. The use of 532 nm wavelength nanosecond pulses for the bottom-up cutting increased the glass-cutting speed up to 9 mm/s [19], but the strength of laser-cut glass remained higher when cutting was conducted in water via direct laser ablation. 

Micromachining with femtosecond pulses in a liquid environment can facilitate the formation of filaments within the water layer [24]. Laser beam filamentation can occur in the water layer when the peak pulse power exceeds a critical power *P*_c_ [37]. The refractive index of water is *n* = 1.329, and the nonlinear refractive index is *n*_2_ = 4.1 × 10^−20^ m^2^/W for a 1030 nm wavelength radiation [38]. Thus, in water, the *P*_c_ value is 2.9 MW for 1030 nm wavelength radiation which for 350 fs duration pulses is reached at a pulse energy of 1.1 µJ. Glass cutting and drilling via femtosecond-pulse filamentation in water were successfully employed in [24,39,40]. The strong electron plasma formation and electron relaxation in the filament facilitated material ablation [41]. Furthermore, filaments can sustain a high beam intensity over a longer distance, allowing cutting and drilling of several-millimeter-deep features without laser beam focal plane readjustment [12,42]. An increase in glass drilling and groove formation speeds was reported in [41]. 

Glass cutting and milling with ultrashort pulses were thoroughly studied by multiple groups on different glasses and glass thicknesses [1,13,21,25,28,36,43,44,45]. However, reliable conclusions on how the laser cutting quality, process throughput, and strength of laser-cut glass parts differ at different glass thicknesses cannot be drawn due to the lack of a systematic approach. Furthermore, to the best of our knowledge, no strength measurements were applied for glass cut with femtosecond pulses in water with pulse energies greatly exceeding the critical self-focusing power threshold. 

In this work, we experimentally studied the femtosecond laser cutting of borosilicate glass plates via direct laser ablation with 1030 nm wavelength radiation. Three glass thicknesses were studied: 110, 300, and 550 µm. We compared cutting in ambient air and water in terms of cut-edge quality and sidewall roughness, ablation efficiency, effective glass cutting speed, and characteristic strength of the front and back sides of laser-cut glass strips.

## 2. Materials and Methods

In this study, we used a femtosecond laser FemtoLux 30 (Ekspla), with a central wavelength of 1030 nm and a pulse duration of 350 fs. In the experiments, the pulse repetition rate *f* was adjusted between 0.4 and 1.1 MHz, resulting in a slight variation in the average laser power *P*. The highest average laser power *P*_max_ was 19.3 W at *f* = 0.4 MHz and linearly increased to 21 W at *f* = 1.1 MHz. The power was measured with an Ophir F150(200)A-CM-16 sensor at the sample surface. The thickest investigated glass (thickness *t* = 550 µm) shattered when cutting was conducted in ambient air at *P*_max_. As a result, we additionally conducted cutting experiments at a low pulse repetition rate of 100 kHz in ambient air. We used a pulse picker to obtain such a low pulse repetition rate. The pulse picker picked specific pulses to obtain the requested pulse repetition rate at the expense of the average laser power. Thus, the average laser power at 100 kHz decreased to 1.8–3.3 W (*P*_min_). Experiments were conducted with a laser beam intensity profile similar to Gaussian (linearly polarized, S polarization).

We used borosilicate glass plates with a thickness of 0.11, 0.3, and 0.55 mm as the samples. Glass plates were thoroughly cleaned prior to the laser treatment and subsequently cut into 26 × 5 mm^2^ glass strips. 

**Figure 1:**
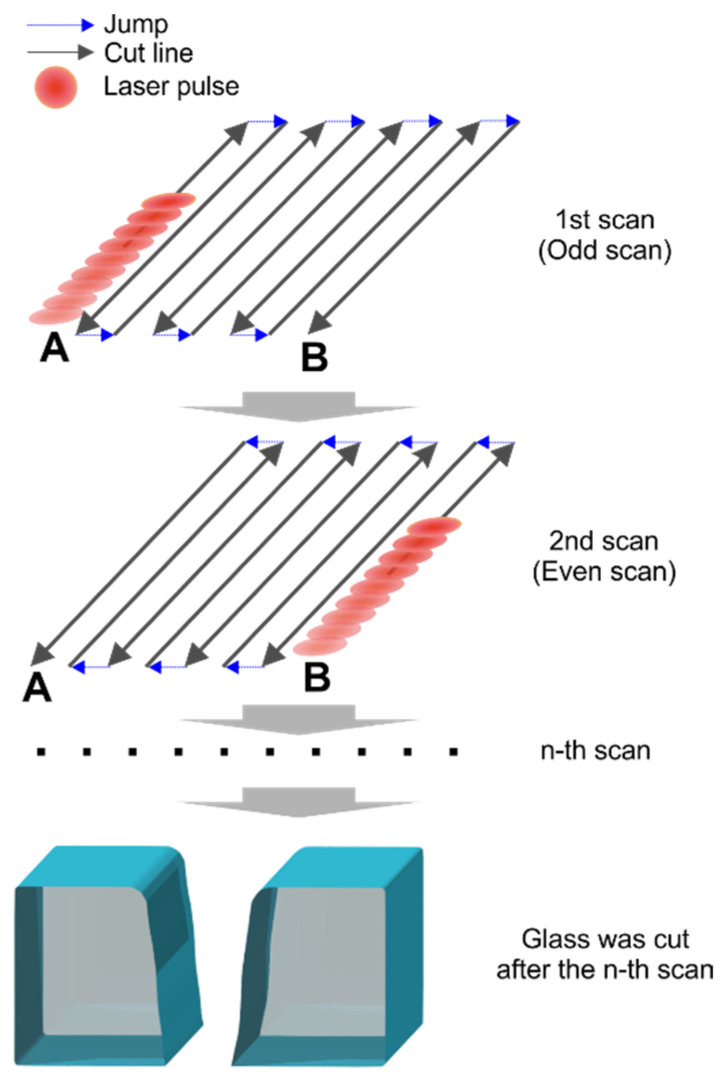
Scan geometry used to cut glass. Arrows represent the laser beam scanning direction for the odd and even scans. For the odd scan, the laser beam scanning direction was from position A to B. For the even scan, the laser beam direction was the opposite (laser beam was scanned from position B to A). Scans were repeated multiple times until the glass was cut through.

Laser cutting was realized with a galvanometer scanner IntelliSCAN_de_14 from ScanLab by scanning parallel cut lines separated by hatch distance (see Figure 1). Each cut line was scanned once per scan. After a fixed number of lines, the hatch and the laser beam scanning directions were changed to the opposite. A positive hatch and scanning direction (A to B) were used for odd scans. A negative hatch with the opposite scanning direction (B to A) was used for even scans. The aforementioned number of cut lines and the hatch distance defined the width of the cut. Scans were repeated multiple times until the glass plate was cut through completely. 

The laser beam was focused to a diffraction-limited spot size (diameter) of 27 μm (in both cutting environments) using an f-theta objective with a focal length of 100 mm. We performed spot-size measurements on thin chrome film deposited on glass plates, according to [46]. The focused beam spot size and the laser fluence values reported in this study were evaluated with a laser beam focal point set at the front surface of the glass sample. For the cutting, the focus was set below the front sample surface at a distance equal to 1/2 the thickness of the glass plate, where the highest cutting speed was achieved in ambient air. Thus, the laser beam focal point was shifted from 55 to 275 μm below the front surface, depending on the glass thickness. 

**Figure 2:**
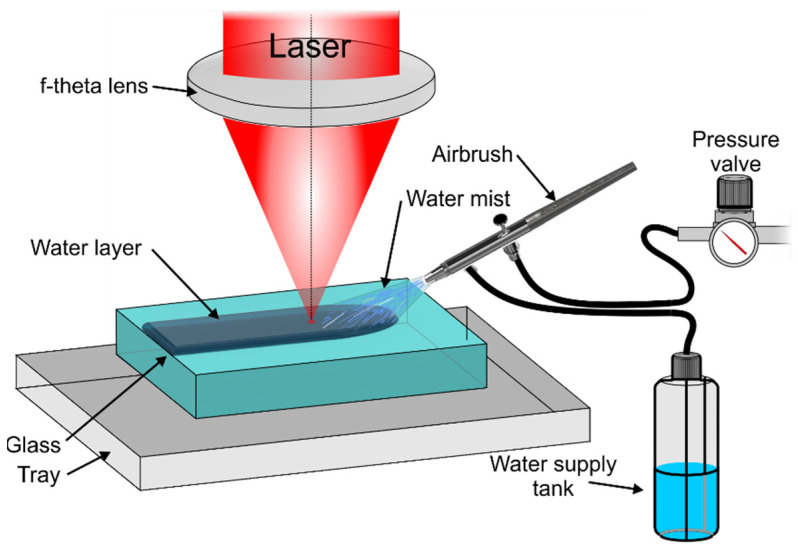
Schematics showing the setup used to form a flowing water layer on top of the glass sample.

In the case of glass cutting in a water environment, cutting was realized through a thin flowing layer of deionized water. The water layer was formed using the water film formation subsystem, which consisted of the compressed air source, an airbrush, a pressure-regulating valve, a deionized water supply tank, and a tray to collect water. The airbrush, connected to pressurized air at 3 bar and a water supply tank, sprayed water mist on top of the glass plate, forming a thin flowing water film (see Figure 2). Constant air pressure and continuously maintained water level in the water tank formed a consistent water film that did not change over time. The nozzle of the airbrush was set 1 cm above the glass surface at an angle of 45 degrees. The liquid flow rate was 11 mL/min. The water mist impingement point was set 5 mm from the laser cutting area (along the water flow direction) to avoid laser beam disturbance with the water mist. The water flow and beam-scanning direction were in parallel. The area covered with the thin flowing water film was 35 mm in length. The width of the film was as wide as the glass plates (the widest plate was 24 mm). The thickness of the water layer decreased linearly with increasing distance from the water mist impingement area. The thickness at the cut line start point was 650 µm, while at the end of the cut, the thickness decreased to 350 µm, giving the average water-layer thickness value of 500 μm throughout the 26 mm long cut line. Initial experiments revealed that the water thickness variation had no significant effect on the ablation efficiency or quality in the laser cutting area. In the experiments, the peak pulse power exceeded the critical power of *P*_c_ = 2.9 MW for the laser beam self-focusing in the water layer from 30 to 40 times, depending on the pulse energy. Butkus et al. [41] simulated the required filament initiation length in water to be 0.5 mm, which coincided with the average water layer thickness used in this study. As a result, we consider that suitable conditions for filament formation in water were ensured. In ambient air, laser ablation was facilitated at the front glass surface, creating favorable conditions for direct material ablation.

After the cutting, glass chipping and cracking were evaluated with an optical microscope Eclipse LV100NDA from Nikon, while cut sidewalls were analyzed with an optical profiler S neox from Sensofar. We used a four-point bending test to determine the maximum bending strength of laser-cut glass strips. The span of supporting and loading rollers was 16 and 6 mm, respectively. Strips were bent from both sides until the failure occurred. We measured the bending strength with a high-precision dynamometer FMI-S30A5 from Alluris. The maximum bending strength was evaluated using the following formula: $\sigma =3F\left(L-l\right)/\left(2bt^{2}\right)$, where *F* is the loading strength at which the glass strip failed, *b* is the width of the strip, *t* is the thickness of the strip, and *L* and *l* are the spans of the support and loading rollers, respectively. The *σ* values obtained were used for the Weibull analysis to extract the characteristic strengths (*σ*_0_) of laser-cut strips. Here, the characteristic strength defines the bending strength at which 63.2% of strips fail. More detailed information on the four-point bending setup and Weibull analysis can be found in [35].

## 3. Results

### 3.1. Optimized Cutting Parameters 

Strips with dimensions of 26 × 5 mm^2^ were cut out of larger glass plates using the laser-cutting parameters presented in Table 1 and Table 2. Glass cutting was performed in ambient air and water. Laser-cutting parameters were optimized, prioritizing the process throughput (effective cutting speed). Here, the effective cutting speed is defined as the ratio between the laser beam scanning speed and the total number of scanning passes (the number of cut lines in a single scan multiplied by the number of scans). The laser fluence *F*, laser beam scanning speed *v*, hatch, number of scans, and cut widths were individually optimized for glass plate thickness and cutting environment during initial experiments. 

The highest effective cutting speed in ambient air was obtained when the focus position was shifted below the front glass surface at a distance of *t*/2, where *t* is the glass thickness. We used the same focus positions for cutting in water as in the ambient air since the ablation efficiency in water was insensitive to focus variation in the *z* direction (in the *z* range between *z* = 0 and *z* = *t*). 

Also, we used a fixed-beam focus position for glass cutting—the Rayleigh distance in ambient air (560 μm) was longer than the *t* of all investigated glass plates. 

In ambient air, 550 µm thick glass plates shattered during cutting at full laser power (*P*_max_) due to excessive stresses caused by heat accumulation. Thus, only *t* = 110 and 300 μm thickness glass strips were cut without shattering into smaller pieces. For this reason, cutting experiments in ambient air were split into two separate cutting regimes: low laser power (*P*_min_, where *f* was limited to 100 kHz (*f*_min_)) and high laser power (*P*_max_, where the maximum pulse rate *f*_max_ was used). In the high-laser-power regime, the applied pulse repetition rate was determined by the maximum laser power (at a given *f*) and laser pulse energy at which optimal fluence was reached. As was mentioned in Section 2, the average laser power at *P*_max_ was distributed between 19.3 and 21 W, depending on the pulse repetition rate. In the low-laser-power regime, the incident laser power was decreased to 1.8–3.3 W by limiting the pulse repetition rate to 100 kHz (*f*_min_) but maintaining optimal laser fluence. 

Almost 200 stripes were cut and investigated in this study (eight cutting regimes × 24 glass strips) in terms of cut-edge quality, cut-sidewall roughness, and characteristic strength. More than 70 strips were cut in water, while 120 strips were cut in ambient air.

### 3.2. Cutting Quality

In this section, we assessed the cutting quality of laser-cut glass strips (26 × 5 mm^2^) in terms of (1) cut-sidewall steepness, (2) maximum defect width, (3) mean defect width, and (4) cut-wall roughness. Cutting quality at the front and back sides of laser-cut glass strips was evaluated separately. 

**Figure 3:**
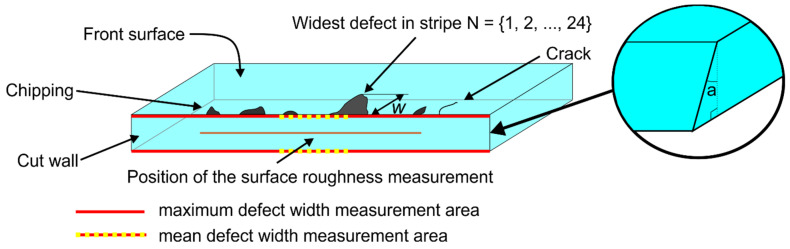
Schematic for evaluating the mean and maximum defect widths. Position for the cut-sidewall roughness measurements and cut-sidewall taper angle (*a*) are indicated.

The cut-wall steepness *a* (the taper angle) was evaluated with an optical microscope. A schematic is shown in Figure 3. According to the results, the steepness of the cut sidewalls ablated in water increased with glass thickness from 13.1° to 10.7° with an average taper angle value of 11.9 ± 0.04°. The taper angle in ambient air (insignificant difference between power levels) was much higher at all investigated glass thicknesses: at *t* = 110 µm the angle was 27.5 ± 2.6°, at *t* = 300 µm it was 18.9 ± 0.6°, and at *t* = 550 μm, the taper angle decreased to 17.3 ± 1°.

The formation of steeper angles in water was conformed in other studies [20,35,47]. Steeper sidewalls (smaller taper angle) allow the maintenance of a higher fluence at greater cut depths due to a flatter bottom of the ablated feature. When cutting in ambient air, a more pronounced V-shape forces one to widen the cut to maintain a high ablation efficiency [20,48]. Otherwise, the flat bottom of the ablated channel transforms into a V-shape quicker than in water-assisted ablation and leads to a larger laser beam impingement area. This, as a result, reduces the laser fluence falling at the glass surface and leads to a quicker loss of material removal rate. The depth of the cut could even saturate. Therefore, in this study, wider cut widths in ambient air achieved an 18.3% higher ablation efficiency than in water (see Table 1 and Table 2) at the expense of producing wider cuts by 38%. 

Due to steeper cut sidewalls, cuts produced in water could be narrower than in ambient air while maintaining a sufficient ablation efficiency. Therefore, despite the ablation efficiency in water being lower, the actual effective cutting speed was almost identical at *t* = 110 μm (lower by 2%) and already surpassed cutting in ambient air by 25% at a glass thickness of 300 µm. 

The lower effective glass-cutting speed in water at *t* = 110 µm (compared to cutting in ambient air) could be affected by the laser fluence loss in water (laser beam reflections, distortion, scattering, absorption in the water layer), and also due to the increased glass cooling effect in the ablation zone [49,50,51,52]. At greater depths in water, steeper cut walls and flatter groove bottoms mitigated efficiency losses in water. 

Next, we evaluated cut-edge defects at the front and back sides of laser-cut glass strips. Chipping or crack formation from the cut edge were considered defects. The mean defect width *w*_mean_ was evaluated by calculating the average width of every chipping and cracking at the cut edge measured over a distance of 1.5 mm. The measurement area was positioned at the center of the laser cut. The maximum defect width *w*_max_ was identified as the widest defect per single cut edge along the entire length of the laser cut. The width of defects *w* was measured normal to the glass surface, as shown in Figure 3. Each strip consisted of four cutting edges (two at the front and two at the back). All laser-cut strips were measured for *w*_mean_, *w*_max_, and cut-sidewall roughness *R*_a_. Values obtained from strips cut under the same cutting parameters were averaged. Cut-edge quality was evaluated separately for the front and back sides. 

**Figure 4:**
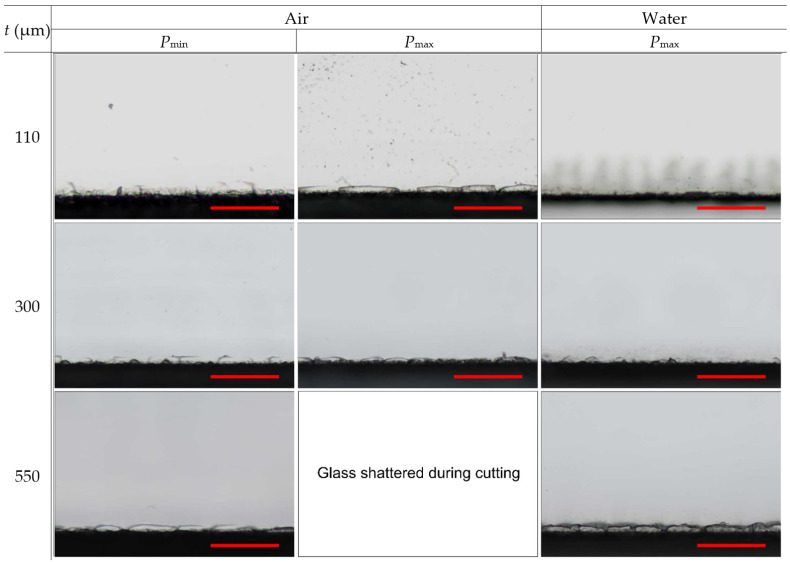
Optical micrographs showing cut edges at the front side of laser-cut glass strips. Rows represent different glass thicknesses, while columns represent different processing conditions. The scale bars represent 100 µm and apply to all panels in the figure.

The typical cut-edge quality at the front glass side is presented in Figure 4. According to the micrographs, the different cutting conditions (cutting environment, glass thickness, and applied laser parameters) had only a little effect on the visual cut quality. Here, the width of the largest defects remained relatively constant despite different laser processing parameters or cutting environments. Only the density and length (along the cut line) of large defects increased with increasing glass thickness, by applying higher laser power, or both. As a result, the maximum defect widths at the front side varied in a narrow range from 13.9 ± 3.8 µm (in water) to 15.5 ± 3 µm (in ambient air at *P*_max_) with an average value of 14.8 ± 0.8 µm (see Table 3). In the table, we present the values for the maximum defect widths averaged over the different glass thicknesses since the dependency on the glass thickness was insignificant. 

**Figure 5:**
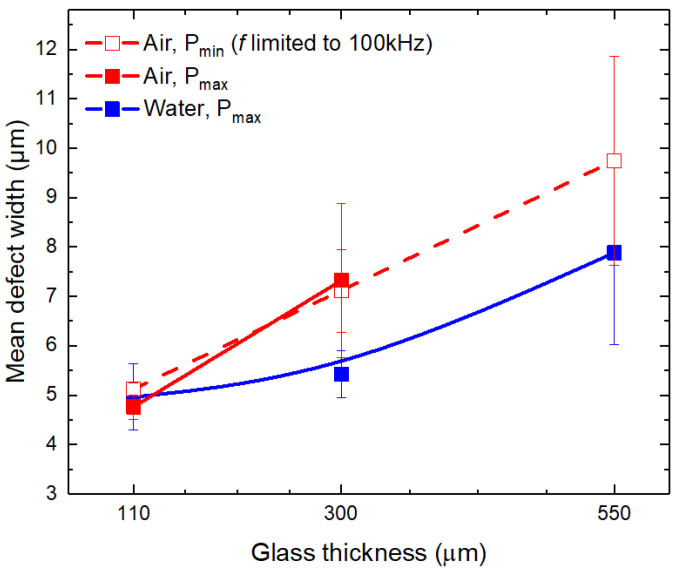
Mean defect widths at the cut edge at the front side of laser-cut glass strips. Dots are connected to guide the eye. Error bars indicate standard deviation.

Contrary to the consistency of *w*_max_, the defects with widths below 10 μm increased with the glass thickness almost linearly. As a result, such development contributed to the increase in *w*_mean_ in ambient air and water environments (see Figure 5). 

At *t* = 110 μm, the mean defect width was almost identical between the different cutting regimes with the average mean defect value of 4.95 ± 0.2 μm (ambient air at *P*_max_ and *P*_min_, and water). However, the mean defect width at higher glass thicknesses was narrower in water: at *t* = 300 μm it was 25% lower (5.4 ± 0.5 μm versus 7.2 ± 1.2 μm) and 19% lower at *t* = 550 μm (7.9 ± 1.9 μm versus 9.8 ± 2.1 μm) than in cuts produced in ambient air. 

**Figure 6:**
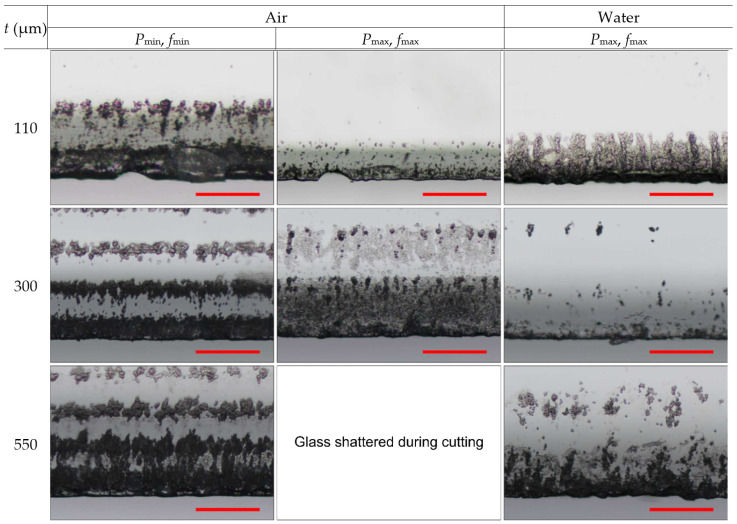
Optical micrographs showing cut edges at the back side of laser-cut glass strips. Rows represent different glass thicknesses, while columns represent different processing conditions. The scale bars represent 100 µm and apply to all panels in the figure.

Cut-edge quality at the back side is presented in Figure 6. Micrographs indicated the damage at the back side consisting of defects at the cut edge (chipping and cracking) and periodically recurring band-like damage expanding further away from the cut edge. 

**Figure 7:**
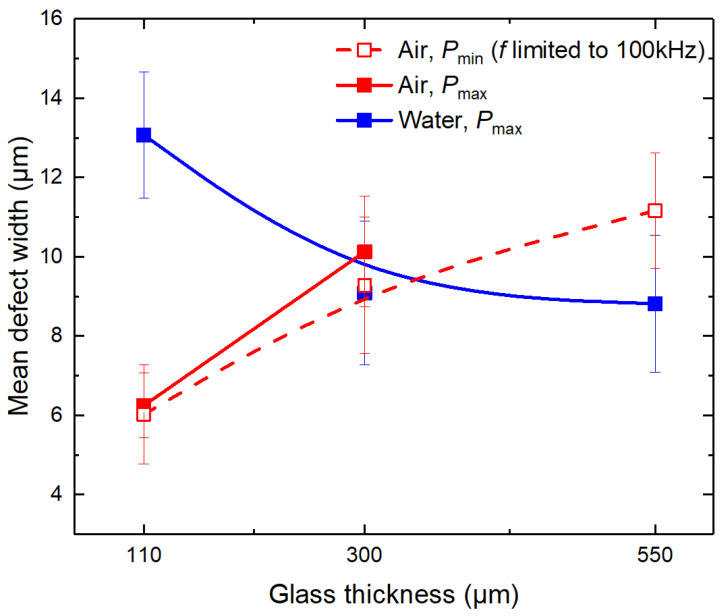
Mean defect widths at the cut edge at the back side of laser-cut glass strips. Dots are connected to guide the eye. Error bars indicate the standard deviation.

The mean defect width’s dependency on the glass thickness in ambient air and water was the opposite (see Figure 7). In the ambient air, the mean defect width increased with the glass thickness. At t = 110 μm, cutting in ambient air at two different power levels (*P*_min_ and *P*_max_) produced cuts with almost identical mean defect width values (average *w*_mean_ value was 6.1 ± 0.2 μm). However, the transition to *t* = 300 μm showed wider mean defects in strips cut at high laser power (10.1 ± 1.4 μm versus 9.3 ± 1.7 μm), indicating a more pronounced heat accumulation in the glass. Finally, the mean defect width at *t* = 550 μm and *P*_min_ reached the highest value of 11.2 ± 1.4 μm. No data at *t* = 550 μm and *P*_max_ were available in ambient air due to glass breaking.

Contrary to the results obtained in ambient air, the mean defect width in water decreased with glass thickness. The highest mean defect width (13.1 ± 1.6 μm) was measured in the thinnest glass strips. However, the *w*_mean_ rapidly decreased: at *t* = 300 μm, the mean defect width was 9.1 ± 1.8 μm, while at *t* = 550 μm, it decreased further to 8.8 ± 1.7 μm. At *t* = 300 μm, the cut-edge quality surpassed cutting in ambient air in terms of mean defect width. 

On average, the maximum defect width at the back surface was 1.8 times larger than on the front side (Table 3). The smallest value of 24.2 ± 12 µm was measured in strips cut in ambient air at *P*_min_. Transitioning to high laser power (*P*_max_) in ambient air increased the maximum defect width to 29.9 ± 5.5 µm. Cutting in water produced a maximum defect width of 25 ± 7.5 µm, which was similar to that measured in ambient air at *P*_min_.

**Figure 8:**
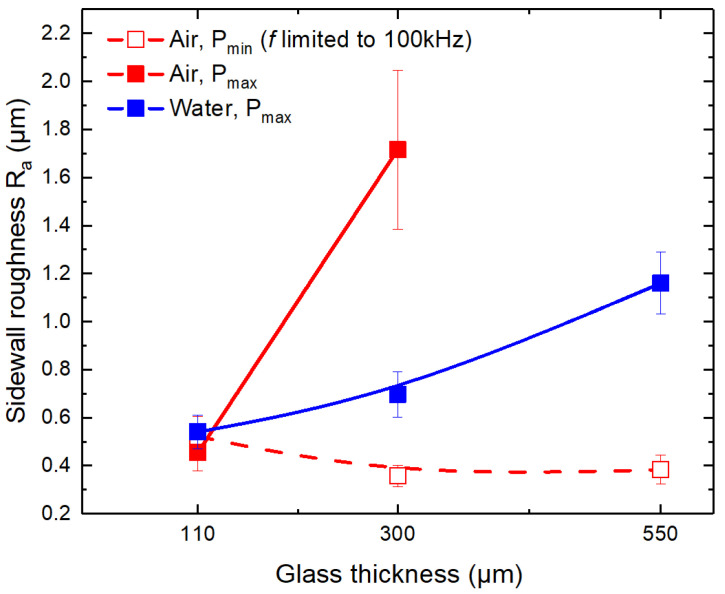
Cut-sidewall roughness versus glass thickness. Glass strips were cut in ambient air and water environments. Dots are connected to guide the eye, while error bars indicate the standard deviation.

Cut-sidewall roughness *R*_a_’s dependency on the glass thickness is presented in Figure 8. According to the results, the sidewall roughness was almost identical at *t* = 110 µm for glass strips cut in ambient air (at *P*_min_ and *P*_max_) and water (at *P*_max_), with an average value of 0.5 ± 0.04 µm. 

In the case of glass cutting in ambient air at limited laser power (*P*_min_), glass thickness had an insignificant effect on the cut-sidewall roughness with an average value of *R*_a_ = 0.42 ± 0.09 µm. In the case of cutting at *P*_max_, the roughness rapidly increased with glass thickness in both ambient air and water environments. In ambient air, the sharp increase in sidewall roughness (from 0.45 ± 0.1 µm at *t* = 110 µm to 1.7 ± 0.3 µm at *t* = 300 µm) indicated significant heat accumulation effects in the laser ablation zone. Cutting in water improved cooling; thus, the increase in sidewall roughness was not as significant as in ambient air at *P*_max_: 0.7 ± 0.09 µm at *t* =300 µm and 1.2 ± 0.1 µm at 550 µm. 

Nevertheless, cutting in water usually results in higher cut-sidewall roughness compared to cutting in ambient air [35,36]. This is associated with increased mechanical glass erosion in water due to plasma and cavitation bubbles generating shockwaves [20,36,53]. Therefore, even if the increasing heat accumulation could be further suppressed in water, the sidewall roughness would remain higher than in ambient air at *P*_min_. 

### 3.3. Band-like Damage

The formation of band-like damage (parallel to the cut edge) at the back surface of transparent media was reported in multiple studies [1,12,54,55]. The cause of the damage was usually associated with laser beam refraction from the sidewall, diffraction from the inverse aperture (the edge of the ablated channel), and multiple reflections between the back and front surfaces of the transparent media.

**Figure 9:**
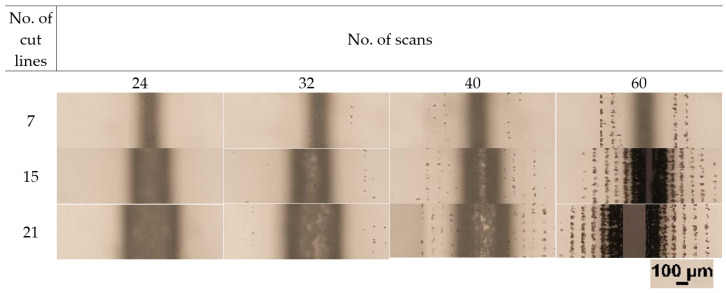
Optical micrographs showing back surface quality after the laser ablation. Rows represent the number of cut lines in a single scan, while columns represent the number of scans. The scale bar shown at the bottom right applies to all the panels in the figure.

In this section, we show that the refraction from the cut sidewall was the main effect causing the band-like damage in laser-cut strips. For this, we produced additional cuts in *t* = 550 µm glass plates in ambient air. The depth and width of cuts were controlled by varying the number of scans (from 24 to 60) and the number of cut lines in a single scan (from 7 to 21), respectively. The hatch distance was fixed at 25 µm. The micrographs showing the back surface of glass plates after the ablation are presented in Figure 9. According to the results, the back side damage occurred after 32 scans. The damage appeared as separate dots clustering into bands. The number of bands increased with the number of scans as the damage accumulated due to repetitive laser beam scanning. Also, the number of individual spots increased and merged into continuous bands after 60 scans. 

**Figure 10:**
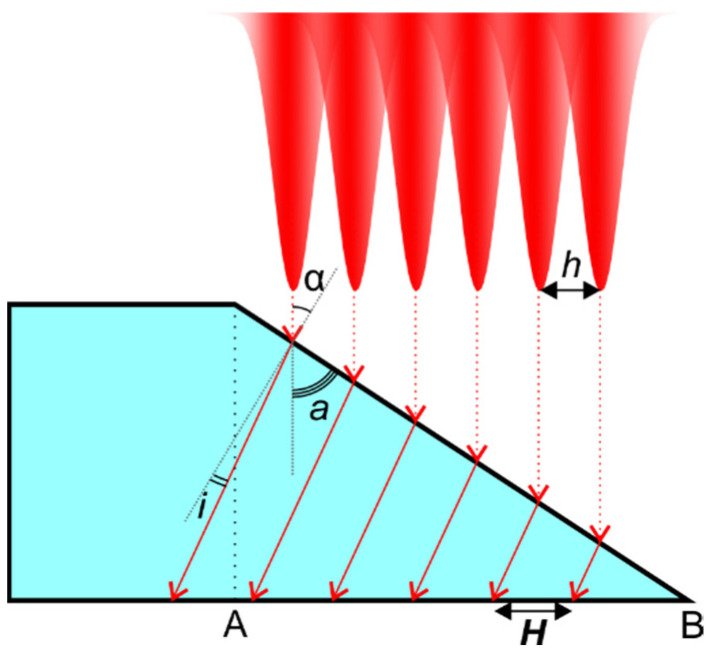
Schematic of the side view of the glass plate indicating a cumulative laser intensity profile falling on the channel (cut) sidewall, laser beam refraction, and impingement onto the back surface. Here, every scan consisted of multiple cut lines separated by the hatch distance *h*. Every cut line was scanned once per single scan.

The laser beam scanning geometry used (see Figure 1) with strictly fixed cut line positions led to the formation of the cumulative beam intensity on the cut sidewall, as shown in Figure 10. The damage at the back surface was associated with the laser beam refraction from the channel sidewall onto the back surface of the glass plate. Thus, the interband distance could be determined using the laser beam refraction angle, taper angle, and hatch distance:

(1) $$H=\frac{h\cdot \cos\left(i\right)}{\sin\left(a\right)\sin\left(i+a\right)}$$

Here, *H* is the distance between the bands, *h* is the hatch, *i* is the laser beam refraction angle, and *a* is the taper angle. Snell’s law was used to determine the angle of the refraction based on the laser beam incident angle α and refraction indices of glass and ambient air (cutting environment). Furthermore, the formation of such a laser beam profile led to wavy cut sidewalls consisting of ridges and concavities, which were discussed in more detail in [35]. Therefore, the formation of concavities could act as additional focusing elements affecting the laser fluence of the refracted laser beam.

The measured and calculated interband distances for a different number of scan lines coincided well (Table 4). Measurements and calculations were applied for glass strips cut under laser parameters presented in Table 1 in ambient air, and a high level of coincidence was also observed. 

Figure 6 indicates that the band-like damage in laser-cut glass strips formed in both cutting environments. Most significant damage occurred at the cut’s edge and decreased with increasing distance from the cut. The damage intensity was highest in strips cut in ambient air at low laser power. However, cutting at high laser power contributed to a lesser number of bands and damage intensity at the back surface. The band-like damage was further mitigated in strips cut in water. Sun et al. [1] reported that the band-like damage at the back surface could be reduced by immersing the back surface of the glass workpiece in the water due to weakened interference at the glass surface (the surface which is in contact with the water layer). In our case, the water layer was formed only at the front surface of the glass plates, keeping the back surface dry. In addition, we observed decreased damage in strips cut in ambient air at high laser power. Therefore, decreased band-like damage intensity in water and ambient air at high laser power was mainly associated with the increased formation and shielding of plasma and laser beam scattering in water. We believe that higher sidewall roughness observed in thicker glasses (300–500 µm) contributed to the scattering of the laser beam (see Figure 8). 

**Figure 11:**
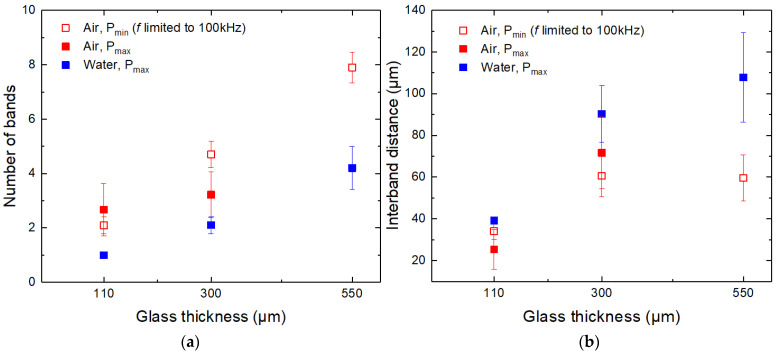
Number of damage bands (**a**) and distance between the bands (**b**) versus glass thickness in water and ambient air cutting environments. Error bars indicate the standard deviation.

Figure 11a shows that the number of damage bands formed at the back surface increased with the glass thickness. The main contributor to the increase was the cut-sidewall protrusion length (the distance between A and B points in Figure 10). Sidewall protrusion length increased with glass thickness due to the taper angle, determining the number of cut lines projected on the cut sidewall. In ambient air, the number of bands was between two and eight in the 110–550 µm glass thickness range. Fewer bands were formed in water due to the steeper cut sidewalls (one to four bands, depending on the glass thickness). 

Figure 11b shows the relationship between the interband distance and the glass thickness. Here, the steepness of the cut wall (taper angle), the hatch distance, and the laser beam refraction angle determined the interband distance. In our case, steeper sidewalls obtained in water contributed to greater interband distances than in ambient air.

Using liquids with other refraction indexes than water would affect the laser beam refraction angle from the cut sidewall. In the case of liquids with a refraction index similar to glass, the refraction angle should decrease, shifting damage bands closer to the cut edge. This would concentrate the back side damage into a smaller area, but the number of damage bands should remain the same. However, it is unclear how the concentrated damage at the edge of the cut could affect the flexural strength of laser-cut glass strips.

### 3.4. Flexural Strength Measurements and Analysis

In this section, we investigated the characteristic strength of laser-cut glass strips (26 × 5 mm^2^) cut in ambient air and water environments. Laser-cut glass strips were broken using the four-point bending setup described in Section 2 and [35]. The front and back side characteristic strengths were evaluated. Characteristic strength measurements for glass strips with dimensions of 26 × 5 mm^2^ were conducted for *t* = 300 and 550 μm glass strips. The strength of the thinnest glass plates of *t* = 110 μm was evaluated by cutting and breaking wider 26 × 20 mm^2^ strips. The width of the strips was increased to ensure sufficient sensitivity and reliability of the breaking force measurement system by increasing the force required to break the glass strips. 

**Figure 12:**
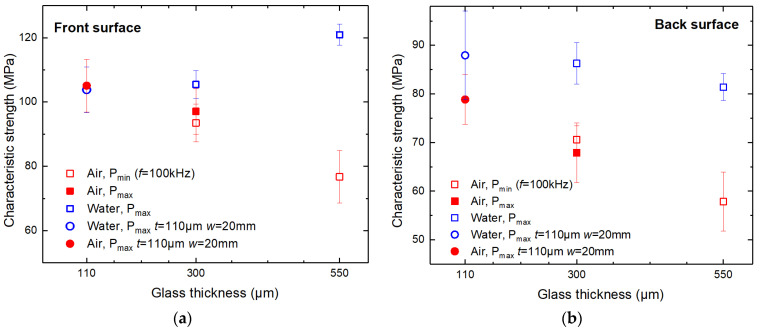
Characteristic strength of laser-cut glass strips. The bending force was applied from the front (**a**) and back (**b**) sides.

According to the measurements presented in Figure 12, the front side characteristic strength in both cutting environments was, on average, 33% higher than at the back side.

We observed that the front side strength dependence on the glass thickness was opposite in strips cut in ambient air and water. In the case of the thinnest glass strips (*t* = 110 µm, *w* = 20 mm wide strips), the characteristic strength at the front side was almost equal between the two cutting environments: cutting in ambient air resulted in a higher front side strength by only 1.3% (103.8 ± 7 versus 105.1 ± 8 MPa). However, as the glass thickness increased, cutting in ambient air became inferior to cutting in water in terms of strip strength. The front side characteristic strength at *t* = 300 µm was higher in water by 8.6–12.8% than in ambient air (105.5 ± 4.3 MPa in water, 97.1 ± 7 MPa and 93.5 ± 6 MPa in ambient air at *P*_max_ and *P*_min_, respectively). The difference increased further at *t* = 550 µm, reaching 58% (121 ± 3 versus 76.7 ± 8 MPa). 

The strength of strips cut in the ambient air at two different power levels demonstrated similar results. Cutting 300 µm thick glass strips at *P*_max_ (19.3 W) and *P*_min_ (3.2 W) resulted in front side glass strip strengths of 97 ± 7.1 and 93.5 ± 6 MPa, respectively. Here, the difference in characteristic strengths was negligible—only 3.6 MPa—and was smaller than the calculated standard deviations of both values (similarly in Figure 12b). 

The back side strength of strips cut in both environments decreased with increasing glass thickness (see Figure 12b). However, cutting in water produced strips with a higher characteristic strength. Also, glass strips cut in water lost strength less rapidly than strips cut in ambient air. As a result, the absolute characteristic strength difference at the back side between the two environments increased with glass thickness: at *t* = 110 µm, the difference was 9.3 MPa (87.9 ± 9.1 MPa versus 78.6 ± 5.1 MPa) and reached 23.5 MPa at *t* = 550 µm (81.4 ± 2.8 MPa versus 57.9 ± 4.7 MPa). 

In Section 3.2 and Section 3.3, we investigated cutting quality. The mean and maximum defect width and glass surface damage analysis revealed that in terms of cutting quality, glass cutting in water was superior for glass thicknesses of ≥300 µm. Mean defect widths at the front (Figure 5) and back sides (Figure 7) of laser-cut glass strips were smaller when cutting was performed in water. Also, the number of bands and the intensity of damage at the back surface were reduced (Figure 6 and Figure 11a). Lastly, the maximum defect width at the back side was smaller by 16.4% at the same laser power level as in ambient air (P_max_, see Table 3). 

The improved overall cutting quality in water at t = 300 and 550 µm led to higher characteristic strength than cutting in ambient air. The most significant improvement was observed at 550 µm glass thickness, where the front and back side strengths were higher by 58 and 40%, respectively. 

On the other hand, cut-sidewall roughness measurements contradicted the characteristic strength results (Figure 8). Cutting in water produced a sidewall roughness up to three times higher compared to cutting in ambient air at *P*_min_ at *t* = 550 µm. Despite that, the strength of strips cut in water was significantly higher than in ambient air. Furthermore, the *R*_a_ was distributed in a relatively wide range (from 0.36 ± 0.04 up to 1.72 ± 0.3 µm) at *t* = 300 µm when cutting was conducted in ambient air at *P*_min_ and *P*_max_. Despite the difference in sidewall roughness, the characteristic strengths of strips cut in ambient air were almost identical at both power levels.

The cut-sidewall roughness is reported to be related to the strength of the glass strips, where an increase in sidewall roughness could lead to strength degradation [45,56]. However, we did not observe such a relationship between glass strength and cut-sidewall roughness in this study. Furthermore, this is supported by our previous studies in [35] (borosilicate glass cutting with 355 nm wavelength ps pulses) and [36] (soda–lime cutting with 1064 nm wavelength ps pulses). In all cases, the cut-wall roughness and characteristic strength were higher in strips cut in water. Thus, we speculate that the sidewall roughness has an insignificant influence on the strength of laser-cut glass strips, at least for low *R*_a_ values. 

However, a similar cutting quality, characteristic strength, and effective cutting speed were achieved in both cutting environments at a glass thickness of 110 µm. The almost identical front side characteristic strength (the difference was 1.3% only) in water and ambient air was supported with similar *w*_mean_ and *w*_max_ defect widths at the front side and sidewall roughness in both cutting environments. However, contradictory results were achieved on the back side. The characteristic strength at the back side was lower in strips cut in ambient air at *P*_max_ by 11.5% despite smaller *w*_mean_, less intense band-like damage formation, and lower sidewall roughness. Only the higher *w*_max_ supported the lower back side strength of strips cut in ambient air.

The ability to introduce high average laser power into the material is crucial for achieving high cutting speeds, especially for direct material ablation and cutting via filament techniques. In this study, the water layer formed on top of the glass plate ensured sufficient glass cooling for all three investigated glass thicknesses, preventing the destruction of glass parts during cutting. For this reason, we only investigated glass cutting in water at the high laser power level. However, based on the previous study on the cutting of borosilicate glass in water with 355 nm wavelength picosecond pulses [35], an increase in incident laser power from 2.8 W to 15.5 W (*P*_max_/*P*_min_ ratio was about 5.5) negatively affected the front cut-edge quality (mean defect width increased from 0.75 to 1.5 µm while maximum defect width increased from 10.5 to 20 µm) and increased cut-sidewall roughness by 20%. As a result, the front side strength of strips cut at 15.5 W decreased by 8.5% compared to cutting at 2.8 W. On the other hand, the overall cutting quality at the back side improved at 15.5 W, but only the front side strength was evaluated. 

We believe that similar changes in cutting quality and characteristic strength could also occur for fs duration pulses when transitioning from several to several tens of Watts of incident laser power.

## 4. Conclusions

In this work, three different thicknesses of borosilicate glass plates (110, 300, and 550 µm) were cut with femtosecond duration pulses into 26 mm long glass strips in ambient air and water. The peak pulse power exceeded the critical power *P*_c_ = 2.9 MW in water by 30 to 40 times, enabling glass cutting via filament formation when a thin water layer was applied on top of the glass plates. Cutting in water and ambient air was investigated.

The maximum power (*P*_max_) of the femtosecond laser was used for cutting glass in water (19.3–19.5 W). Only the two glass plates (110 and 300 µm) could be cut into smaller strips in ambient air, while the thickest glass plate (550 µm thickness) suffered from detrimental damage—glass shattering into smaller pieces. For this reason, we conducted glass cutting at high (*P*_max_, 20.8–21 W for 110 and 300 µm thickness glasses) and low (*P*_min_, 1.8–3.3 W for all three glass thicknesses) laser powers in ambient air. 

The analysis of the band-like damage formation at the back surface of laser-cut strips showed that the refraction of the laser beam from the cut sidewall was responsible for the damage formation at the back surface. The distance between damage bands was evaluated based on the laser beam refraction, cut-sidewall taper angles, and hatch distance. 

Experiments revealed that cutting in water was superior for 300 and 550 µm thickness glasses in terms of overall cut-edge quality (mean defect width), effective cutting speed, characteristic strength, and lesser band-like damage at the back surface. Furthermore, the advantage of cutting in water increased with the glass thickness. In the case of ultrathin glass (glass thickness 110 µm), the cutting performance, quality, and characteristic strength were similar in both cutting environments. Thus, cutting ultrathin glass in ambient air might be more attractive due to the simpler laser system setup. 

## Figures and Tables

**Table 1:** Glass cutting parameters and performance for fs cutting in ambient air. Values presented outside brackets represent cutting at high laser power (P_max_), while values inside brackets represent cutting at low laser power (P_min_). Cutting width, hatch, and the number of cut lines were maintained the same for both high- and low-power cutting.

Glass Thickness (μm)	Average Laser Power (W)	Pulse Repetition Rate (kHz)	Scanning Speed (mm/s)	Number of Cut Lines in a Single Scan	Fluence (J/cm^2^)	Hatch (μm)	Cut Width (μm)	Ablation Efficiency (μm^3^/μJ)	Effective Cutting Speed (mm/s)
110	21 (1.8)	1100 (100)	1600 (150)	7	6.7 (6.3)	20	150	10.6 (11.3)	20.8 (2)
300	20.8 (3.2)	620 (100)	1000 (170)	13	11.7 (11.2)	22.5	310	8.6 (8.8)	3.2 (0.5)
550	- (3.3)	- (100)	- (250)	17	- (11.5)	20	350	- (7.9)	- (0.17)

**Table 2:** Glass-cutting parameters and performance for fs cutting in water. Cutting in water was conducted at a high laser power P_max_ only.

Glass Thickness (μm)	Average Laser Power (W)	Pulse Repetition Rate (kHz)	Scanning Speed (mm/s)	Number of Cut Lines in a Single Scan	Fluence (J/cm^2^)	Hatch (μm)	Cut Width (μm)	Ablation Efficiency (μm^3^/μJ)	Effective Cutting Speed (mm/s)
110	19.5	530	1100	9	12.9	12.5	135	8.7	20.4
300	19.3	433	500	9	15.6	22.5	210	7.2	4
550	19.3	433	500	11	15.6	22.5	260	7.3	1.8

**Table 3:** Maximum defect widths averaged over different glass thicknesses (110, 300, and 550 µm). Cases for the front and back sides are presented separately.

Cutting Regime	*w*_max_ at the Front Side	*w*_max_ at the Back Side
Air (*P*_min_, *f*_min_)	15 ± 3.4 µm	24.2 ± 12 µm
Air (*P*_max_, *f*_max_)	15.5 ± 3 µm	29.9 ± 5.5 µm
Water (*P*_max_, *f*_max_)	13.9 ± 3.8 µm	25 ± 7.5 µm

**Table 4:** The distance (period) between the individual band-like damages formed during glass cutting in ambient air.

Hatch (µm)	No. of Cut Lines	Period between Damage-like Bands	
		Calculated (µm)	Measured (µm)
25	7	89.9	86.4
25	15	79.3	82.8
25	21	88.4	87.7

## Data Availability

The data presented in this study are available on request from the corresponding author.
